# The association between social support and physical activity in older adults: a systematic review

**DOI:** 10.1186/s12966-017-0509-8

**Published:** 2017-04-27

**Authors:** Gabrielle Lindsay Smith, Lauren Banting, Rochelle Eime, Grant O’Sullivan, Jannique G. Z. van Uffelen

**Affiliations:** 1 Institute of Sport, Exercise and Active Living, Victoria University, Melbourne, Australia; 2 Faculty of Health, Federation University, Ballarat, Australia; 3 Department of Kinesiology, Physical Activity, Sports and Health Research Group, KU Leuven - University of Leuven, B-3000 Leuven, Belgium

**Keywords:** Physical activity, Social support, Loneliness, Older adults/aging, Systematic review

## Abstract

**Background:**

The promotion of active and healthy ageing is becoming increasingly important as the population ages. Physical activity (PA) significantly reduces all-cause mortality and contributes to the prevention of many chronic illnesses. However, the proportion of people globally who are active enough to gain these health benefits is low and decreases with age. Social support (SS) is a social determinant of health that may improve PA in older adults, but the association has not been systematically reviewed.

This review had three aims: 1) Systematically review and summarise studies examining the association between SS, or loneliness, and PA in older adults; 2) clarify if specific types of SS are positively associated with PA; and 3) investigate whether the association between SS and PA differs between PA domains.

**Methods:**

Quantitative studies examining a relationship between SS, or loneliness, and PA levels in healthy, older adults over 60 were identified using MEDLINE, PSYCInfo, SportDiscus, CINAHL and PubMed, and through reference lists of included studies. Quality of these studies was rated.

**Results:**

This review included 27 papers, of which 22 were cross sectional studies, three were prospective/longitudinal and two were intervention studies. Overall, the study quality was moderate. Four articles examined the relation of PA with general SS, 17 with SS specific to PA (SSPA), and six with loneliness. The results suggest that there is a positive association between SSPA and PA levels in older adults, especially when it comes from family members. No clear associations were identified between general SS, SSPA from friends, or loneliness and PA levels. When measured separately, leisure time PA (LTPA) was associated with SS in a greater percentage of studies than when a number of PA domains were measured together.

**Conclusions:**

The evidence surrounding the relationship between SS, or loneliness, and PA in older adults suggests that people with greater SS for PA are more likely to do LTPA, especially when the SS comes from family members. However, high variability in measurement methods used to assess both SS and PA in included studies made it difficult to compare studies.

**Electronic supplementary material:**

The online version of this article (doi:10.1186/s12966-017-0509-8) contains supplementary material, which is available to authorized users.

## Background

The global population is ageing due to an increase in life expectancy and a reduction in fertility rates. In 2010, an estimated 524 million people were aged 65 or older – 8% of the world’s population. By 2050 this is expected to nearly triple to 1.5 billion, representing about 16% of the world’s population [1]. It is well known that age is an independent risk factor for the development of non-communicable diseases (NCDs) such as cardiovascular disease, cancer, diabetes and dementia [1]. Even without NCDs, function and independence generally decline in older age as a result of reductions in cognitive and physical capacity (e.g. muscle strength, balance, cardiovascular endurance) [2, 3]. Now more than ever it is vital to investigate ways to encourage aging well [4] or ‘Active Ageing’. This phrase refers to older adults being enabled to continue participating in “social, economic, cultural, spiritual and civic affairs” and maintain a good quality of life [5]. Promotion of Active Ageing has the potential to slow the otherwise ever-growing burden on national economies and health care systems worldwide and more importantly, to ensure that older adults are able to enjoy their lives to the best of their capacities.

Performing sufficient physical activity (PA) is a primary modifiable determinant of health especially pertinent to Active Ageing because it is known to have vast mental and physical health benefits for people of all ages [6, 7]. In adults, PA reduces the risk of all-cause mortality, prevents various chronic diseases, and in older adults especially, it reduces the risk of falls and helps maintain physical and cognitive function [8–17]. Despite the known benefits of regular PA [18], 23% of adults globally are insufficiently active, with some high income countries having inactivity rates of up to 54% [14]. Inactivity rates increase with age, with around two-thirds of those between 65-74 years and three-quarters of those over 75 years not meeting PA guidelines of at least 150 min/week of moderate intensity activity in either the US [15] or Australia [17].

In order to have a more active and healthy ageing population, it is vital to investigate ways to increase PA levels in older adults. Research addressing the most appropriate intervention methods is still inconclusive. However, many behaviour change theories, including the Social Cognitive Theory [19], Social-Ecological Model [20, 21], Theory of Planned Behaviour [22] and the Health Belief Model [23], highlight the importance of social factors such as social support (SS) and social connectedness in maintaining and/or initiating behaviour change. These theories have also been used in PA behaviour change research with older adults [24–29]. Furthermore, the World Health Organisation (WHO) identifies SS as a key determinant of Active Ageing [5], because of the importance of strong social ties for life satisfaction and subjective wellbeing in older adults. It is vital that social interactions are maintained with increasing age, as good social functioning is associated with improved self-efficacy [30–32], reduced risk of depression [33, 34] and a reduced risk of all-cause mortality [35]. Older adults have the potential to experience greater levels of loneliness and decreased SS as they encounter significant life events such as retirement, loved ones becoming unwell or passing away, or moving into care [36]. Furthermore, experiencing multiple life events at once is associated with a reduction in physical activity levels in this population group [37].

Despite having featured prominently in research for some time, SS is still a contentious and poorly defined concept, but with agreement that it is multifaceted [38–40]. A critical appraisal of the literature by Williams et al. [40] found 25 variations on the definition of SS in use. Key themes identified in the SS definitions were social relationships that are reciprocal, accessible and reliable and provide any or a combination of supportive resources (e.g. emotional) and distraction from stressors or information [40]. Additionally, the WHO defines SS as being both ‘emotional and practical support characterising good social relations’ and a social determinant of health [41]. In the description of SS by the WHO, there is also referral to an absence of loneliness [5]. Whilst social support and loneliness are not the opposite of one another and one can be lonely without being socially isolated, they have been shown to be directly linked in community-dwelling older adults [34] and thus we have included loneliness in this review.

In the general adult population there has been some suggestion that task-specific SS is more important than general support for maintaining or changing health behaviours [42, 43]. However, for PA behaviour this association does not seem to be as clear-cut, with studies supporting a positive association between PA and both general SS [44–46] and support specific to PA [47–49]. It is possible that similar associations also exist in older adults but these have not been summarised before, therefore this will be addressed in this review. There may also be value in understanding the specific role of different sources of SS (e.g. friends, family or exercise group) and PA levels in older adults. Kouvonen et al. [30] reported that people with high emotional support from their closest significant other, who met PA guidelines, were more likely to still be undertaking adequate PA five years later. Eyler et al. [49] found that high SS from both friends and family was significantly associated with greater PA levels in women. Not only the *type* and *source* of SS may play a role in the association between SS and PA, this association may also differ across the PA *domains* of active transport, active recreation or leisure time PA, household activities and occupational activities [50]. For example, studies synthesised in a recent systematic review of the association between SS and PA in adolescents consistently found a positive association between support from both parents and friends and leisure time PA, whereas the transport domain of PA was only consistently and positively associated with SS from friends [51].

As demonstrated above, the research surrounding SS and PA in adults is varied and therefore difficult to generalise to older adults with certainty. Also, in older adults the literature has not been reviewed and summarised in the past. Given the considerable societal changes occurring with the ageing population and the importance of PA to the health and quality of life in older adults, a review of the research evidence for this population group is warranted. Therefore this review has three aims: 1) systematically review and summarise the studies examining the association between SS, including loneliness as per the WHO definition, and PA in older adults; 2) clarify if any potential associations differ between *types* (e.g. task specific support, general support) or *sources* of support (e.g., support from family, friends or exercise group); and 3) investigate whether the association between SS and PA in older adults differs between specific *PA domains* (LTPA, transport, household, occupational).

## Methods

### Protocol

The Preferred Reporting Items for Systematic Reviews and Meta-Analyses (PRISMA) checklist has been followed to undertake this systematic review [52].

### Study eligibility criteria

Studies examining the association between social support, including loneliness, and physical activity (PA) in older adults and meeting the following criteria were included: 1) Generally healthy, community dwelling older adults with a mean age of at least 60 years, as per the definition of the UN [53] and a minimum age of no less than 50. If the mean age or age range of participants was not clear the paper was excluded; 2) A validated measure of SS with at least two items or a validated measure of loneliness; 3) PA was measured objectively or subjectively using measures with established validity as reported in the individual papers, or with clear face validity [54]. As Terwee et al. [54] state in their review of measurement characteristics of PA questionnaires, face validity is often the most important measurement property of a questionnaire and the relevance of other aspects of validation (e.g. reliability, validity, responsiveness) differs depending on what the scale intends to measure. Therefore, PA questionnaires with clear face validity were included in this review. In addition, PA data needed to be analysed appropriately, i.e. studies that analysed ordinal PA data as a continuous variable were excluded; and 4) Peer reviewed, quantitative studies, regardless of study design, available in English, German, French or Dutch were considered for inclusion.

### Information sources and search

Systematic searches of MEDLINE, PSYCInfo, SportDiscus, CINAHL (via EBSCOHost Megafile premier) and PubMed were conducted in August 2014. No limit to dates of coverage was applied to these searches. Free terms as well as appropriate thesaurus terms of each database were combined for the population, SS, including loneliness, and PA. An example full search strategy for PubMed is included in a separate file (see Additional file 1). Full search strategies for EBSCOHost are available from the first author on request.

### Study selection

Results of the database searches were imported into Endnote X7 and duplicates were removed. Titles and abstracts were then screened by one reviewer (GLS) to remove papers out of scope. Next, full texts were screened in detail by one reviewer (GLS) to check if the inclusion criteria were met. Two authors then independently reviewed the papers in the final list. Reference lists of included papers were screened to identify additional studies meeting the inclusion criteria. In case of any uncertainty during the review process, an additional reviewer was consulted and a consensus decision was made.

### Data extraction

Data were extracted by two reviewers according to the following pre-agreed categories: Country where study was conducted, study design, sample size, participant characteristics (age [mean and range], gender), PA and SS or loneliness measures, results and adjustments in multivariate analyses. See Table 2 and Table 3 for further details. Authors were contacted for more information if there was insufficient detail about validation of the SS/loneliness measure.

### Risk of bias and quality assessment

The Gyorkos risk assessment tool was used to rate the quality of included papers, as it includes items to assess the quality of multiple study designs [55, 56]. All reviewers came to a consensus about the definitions of major and minor flaws for the various study designs as recommended in the rating instructions [56]. See Table 1 for details. In addition to overall study design, quality of SS (or loneliness) and PA variables were assessed for each study. For each study, every item was rated independently by 2 reviewers as ‘yes’, ‘partially met’, ‘no’, ‘can’t tell’ or ‘NA’. Based on these ratings the overall quality was rated by each reviewer as:

- Strong: No major flaws, a few minor flaws - any plausible postulated bias was unlikely to seriously alter the results,
- Moderate: No major flaws, some minor flaws - a plausible bias exists that brought into question the confidence that could be attached to the results,
- Weak: One or more major flaws - a plausible bias existed that seriously weakened confidence in the results.

In case of discrepancies between reviewers in the final quality rating, a third reviewer assessed the study and the three reviewers discussed to resolve the disagreement.

**Table 1:** Definitions of major and minor flaws for this SR

	Experimental studies (clinical trial or community trial)	Longitudinal Observation (cohort study or observational study)	Cross-sectional
Study population: Major	• No Control • Sample size inadequate for power (*n* < 20 per group) • Non-random allocation or randomisation not described	• Not representative of the population of interest. In relation to age, gender AND • Confounders not accounted for • No description of sample	• Not representative of the population of interest. In relation to age, gender AND • Confounders not accounted for. • No description of sample
Minor	• Confounders not completely accounted for • Omission of detail about confounders.	• Not representative of the population of interest. In relation to age, gender • Omission of detail about confounders • Non-random sampling • Sample size inadequate for power (*n* < 10 per variable) or not described (if study *n* < 500)	• Not representative of the population of interest. In relation to age, gender • Confounders not accounted for • Non-random sampling • Sample size inadequate for power (*n* < 10 per variable) or not described (if study *n* < 500)
Intervention/ exposure: Major	• No description of the PA or SS component of the intervention • No measurement of intervention strength or exposure • Intervention <12 weeks	• No measurement of exposure • Poor or no face validity of measurement of exposure	• No measurement of exposure • Poor or no face validity of measurement of exposure
Minor	• No blinding	• No validity of measurement PA or SS exposure mentioned	• No validity of measurement PA or SS exposure mentioned
Outcome: Major	• Poor face validity of measurement of outcome	• Poor or no face validity of measurement of exposure	• Poor or no face validity of measurement of PA or SS outcome
Minor	• No validity of PA or SS outcome measure mentioned	• No validity of PA or SS outcome measure mentioned	• No validity of PA or SS outcome measure mentioned
Follow-up: Major	• High drop-out (>20%) (from pre to post- test measurement)	• High drop-out (>20%) (from pre to post-test measurement)	• NA
Minor	• High drop out in long term follow up (post intervention) • No long term follow up	• High drop out in long term follow up (post intervention) • No long term follow up	• NA

### Data synthesis

The studies were categorised as focusing on loneliness, general SS or SS specific to PA. Each paper was rated as + or - if a statistically significant positive or negative association was found; 0 was assigned if no statistically significant association was found (In the remainder of this paper, ‘statistically significant’ results will be referred to as ‘significant’). Studies reporting differences in results for males and females, or in the type, source and/or domain or PA are coded multiple times (See Table 3 for details of quality assessment). To summarize the associations found in the studies, the following overall ratings, as suggested by Sallis et al. [57], were given to each section: “0” (No association; 0%-33% of the findings supported the association), “?” (indeterminate association; 34-59% of the findings supported the positive or negative association), “+” or “- “(positive or negative association; 60%-100% of the findings supported the association) [57] (see Table 4 for overall quality ratings for each category).

## Results

### Study selection

**Fig. 1:**
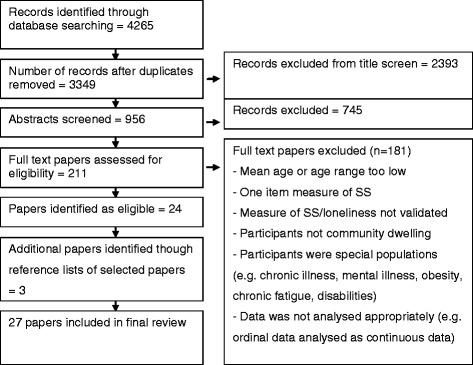
Search process flow chart

Of the 4265 papers identified in the search, 3349 remained after removing duplicates. After screening titles and abstracts to remove papers out of scope, the full text of 211 papers was checked. Of these, 24 met the inclusion criteria. Three further relevant studies were identified in backward reference tracking giving a total of 27 papers for the review (See Fig. 1).

### General study characteristics

More than three quarters of the included studies (21 studies) examined the association between SS and PA, and the remaining six studies investigated the relationship between loneliness and PA levels [58–63]. Of the studies examining the association between SS and PA, 17(81%) examined the association between SS specific to PA or exercise [31, 64–79] and the remaining four examined the association between general SS and PA [80–83].

The majority of the studies (67%) were published between 2006 and 2014 with the oldest paper published in 1992 [83]. Seventeen studies were conducted in either the USA or Canada, with six from Asia, two from Europe [61, 66], one from Australia [64] and one from Israel [59]. More than 80% of the identified studies (22 studies) were cross-sectional, three were longitudinal [58, 60, 71] and two were experimental [66, 79] (see Table 2). Sample sizes ranged from 64 [79] to 13,812 [63]. Most studies incorporated both males and females and four included females only [67,72,78,65]. Only four studies assessed sex differences relating to SS and PA levels [59, 70, 73, 80]. See Table 2 for details.

**Table 2:** Study characteristics: design, study population and measures of SS and PA

First Author (Year) Country	Study design^1^ Sample size^1^	Gender Age (Years): [Range] Mean (SD)	PA Measure^2^	Domain of PA^3^	Definition of ‘active’ ^4^	SS Measure^5^	Type of SS	Source of SS
Booth [95] Australia	XS 402	44.8% male [60+ years] NA	SR: LTPA questionnaire [95]	LTPA (walking and MVPA)	>800 kcal /week spent doing PA	Social environment scale (Likert scale, 4 items). Adapted from Sallis’ SS for ex. Scale [42].	SSPA	friends and family
Bopp [65] Rural USA	XS 102	All female [50+ years] 70.6 (9.2)	SR: PA Scale for the Elderly (PASE) [96]. Strength training.	Strength training exercise (LTPA)		SS for ex. scale [42] Friends 15 qs, family 5 qs. Conflicts with original version (5 friends, 5 family).	SSPA	friends and family
Carlson [31] USA	XS 687-709 (3 PA variables)	46.9% male [65+ years] 74.4 (6.3)	Objective: Accelerometer (1 week) and subjective SR survey: CHAMPS [97]	Total MVPA, TPA		SS for ex. scale. Adapted from [42] & [31]. 4 items; Internal consistency Cronbach’s α = 0.67	SSPA	family and friends (Sum score)
Gellert [66] Germany	Experimental N = 302 *n* = 48 ex. with partner *n* = 84 singles *n* = 170 not ex. with partner	52% male [60-95 years] 66.5 (4.9)	SR PAQ-50 (adapted from [98]. Frequency and duration in last 7 days	HH, LTPA/ sport, TPA		Only 2 items from SS for ex. Scale [42]	SSPA.	friends, partner and family (total)
Hall [67] USA	XS 128	All female [NA] 69.6	Objective: accelerometer analysed as less than 10,000 and more than 10,000 steps per day	All	At least 10 000 steps /day	SS for ex. scale [42] 10 item version, not clear which questions.	SSPA	Friends and family
Kaplan [80] Canada	XS 12611	39% male [65+ years] NA	SR: leisure PA. Monthly moderate-intensity PA lasting more than 15 mins	LTPA	Greater than 15 mins PA at least 12x/m = *frequent;* less than this *= infrequent*	4 questions. Perceived SS [93]. Qs: 1) someone they could confide in, 2) someone they could count on, 3) someone who could give them advice,4) and someone who made them feel loved. Score = sum of all affirmative (yes) responses to the four items. (Internal consistency Cronbach’s α = 0.75.)	General perceived SS. (General SS)	
Kim [68] South Korea	XS 290	30% male [65-89 years] 68.6 (4.4)	SR: Leisure time PA scale [99] Habitual weekly PA. Greater than 15 mins. Scored in METs (sum total of all activities)	LTPA		24 item SS for ex. scale [42]. Translated [100]. 12 friend and 12 family questions. Unclear exactly which Qs.	SSPA	family and friends
Kraithaworn [69] Thailand	XS 258	26% male [60-88 years] 70.0 (6.4)	Modified version of self-reported PAQ for older Thais [101]. 42 items. Number hours /week for each activity. Last 7 days. Scored in METs	All	30 mins mod. intensity PA, 5 days /week or 20 mins vig. PA 3 days /week.	Scale developed from Social support for ex. scale [42] modified version for this study and Thais. 11 items [102]. Unclear which questions used.	SSPA (emotional support, tangible support, informational support.	family and friends
Lian [70] Singapore	XS 2494	42% male [60+ years] NA	SR: Number of times doing moderate or vigorous PA at least 20 mins in last week (moderate: walking, gardening, tai chi chuan, qigong; Vigorous: jogging, cycling, swimming)	LTPA	mod. or vig. PA at least 20 mins at least 3x /week	SS for ex. Scale Adapted from [103]. 6 questions in original; 3 friends, 3 family.	SSPA.	Family and friends
Luo [58] China	Longitudinal. 2002 sample *n* = 14072 re-interviewed 2005 *n* = 7668 re-interviewed 2008 *n* = 4033	47.1% male [65-105 years] 72(6)	SR: Regular PA yes/no?	LTPA		Loneliness (as an IV for health). Single question about loneliness (how often lonely: never - always). 5 point Likert scale	Loneliness	NA
McAuley [71] USA	Longitudinal 153	28% male [60-75 years] 66	PA during the study: attendance at the classes (SR exercise log after each class). Follow up PA at 6 and 18 months assessed by SR: PASE [104] 10 item scale	LTPA, OPA, HHPA, (summed)		Social Support Provisions Scale [105] (support provided by exercise group). 24 items, 6 headings reflecting the social provisions proposed by [106]: attachment, social integration, reassurance of worth, reliable alliance, opportunity for nurturance, and guidance.	SSPA.	Exercise group
Mowen [81] USA	XS 1515	44% male [50-99 years] 67.4(9)	SR: Single ordinal item. Daily PA levels scored 1-3. 1 = sedentary, 2 = moderate activity 3 = vigorous activity (uses examples for each e.g. usually sedentary, usually walking a lot etc.)	LTPA, OPA, TPA (combined)		Social support questionnaire (SSQ) [92]. 6 items. Number of people providing supports and degree of satisfaction with support. 6 Point Likert	General SS, network size, SS satisfaction	Number of people to provide support, satisfaction with. (all people in network)
Netz [59] Israel	XS 1663	48% male [65+ years] 74.8(6.2)	SR: frequency and average time doing certain types of Moderate and vigorous intensity PA each week (e.g. walking etc.).	LTPA	*Sufficiently active:* Mod. PA at least 150 mins /week, or Vig. PA at least 75 mins /week or an equivalent mix of the 2. *Insufficiently active:* Some activity but less than the levels above. *Inactive:* no activity other than light-intensity activity less than 1x /week	1 item from CES-D [107] Lonely/not lonely 4 point Likert scale. A question also about living alone	Loneliness, living alone	NA
Newall [60] USA	Longitudinal 194	37.7% male [77-96 years] 83(4.2)	Objective: Accelerometer. Daily PA	all daily activity		Loneliness measured in 2001 using [108] 11-item loneliness scale.	Loneliness	NA
O'Brien Cousins [72] Canada	XS 327	All female [70-98 years] 76.7 (5.5)	SR: Older adults exercise status inventory (adapted from other scales for this study)	LTPA, OPA, HHPA (summed)		4 Questions (1) previous family sport involvement; (2) current encouragement by at least one person to maintain one's physical abilities; (3) endorsement by the family physician; and 4) peer group interest in physical fitness activity	SSPA	family, friends, doctor (sum score)
Oka [73] Japan	XS 137	47% male [70-89 years] 74.5	Objective: pedometer daily number of steps. Recorded. Over 1 year and data downloaded every month.	All	Men = 6700 steps, women = 5900 steps	Itakura Social support for ex. [109] 5 item scale. Functional, emotional, and informational Social support for exercise i) advice/instruction, ii) understanding/ sympathy, iii) former encouragement/ reinforcement, iv) joint implementation, v) compliment/ appreciation.	SSPA.	family and friends (sum score)
Orsega-Smith [33, 74] USA	XS 1900	38.5% male [50+ years] 67.7 (6.9)	SR: survey frequency, intensity, time of 6 different PAs (specified by participants) over the last week. Total METs calculated.	LTPA	A minimum of 3 METs (mod. level of PA), at least 5 days /week (meeting PA guidelines)	Social support for ex. scale [42] 12 items for friends and family (not clear if 12 is total number of questions or 12 for each friends and family)	SSPA	Family and friends (separate)
Park [75] South Korea	XS 187	29.9% male [57-96 years] 71.6 (5.9)	SR: PASE [110]	LTPA, OPA HHPA (summed)	*high-active =* 150mins mod. and/or 75 mins vig. PA /week. *low-active =* Less than above	Social support for ex scale [42] Not clear if full original questionnaire was used. Translated into Korean.	SSPA	Friends and family
Potts [83] USA	XS. 936	40.4% male [NA] 72.6 (5.9)	SR telephone interview: Regular exercise in last week yes/no	LTPA		Social network scale [91] . 10 items. Number of friends and family (asked separately) seen regularly, able to talk to about private matters and ask for help.	General Social engagement (General SS)	family and friends
Sasidharan [76] USA	XS 1967	Gender NA [50+ years] NA	SR: 5 items from PASE [110]	LTPA		Social support for ex scale [42] 12 questions for each; friends and family	SSPA	friends or family
Schuster [77] USA	XS 108	31% male [60+ years] NA	SR: Frequency of participation in one or more physical activities in last 6 months for 20-30 min?	LTPA		Social support for ex. scale. 5 items. Adapted from Sallis (1987). Not clear which questions	SSPA	Friends and family (combined).
Shankar [61] UK	XS 8688	46.1% male [NA] 66.9 (10.4)	SR: times /week taken part in vigorous, moderate or mild PA	LTPA, OPA	*Inactive =* mod.- vig. PA no more than 1x /week	3 item revised UCLA loneliness scale [111]. And also a measure of social isolation	Loneliness and social isolation	NA
Shiovitz-Ezra [62] USA	XS 3005	46.6% male [57-85 years] NA	SR interview: frequency participating in PA (examples provided), 5-point Likert scale ranging from more than 3x /week to never.	LTPA		Loneliness question from CES-D	Loneliness	Loneliness
Theeke [63] USA	XS 13812	38.7% male [NA] 67.7 (9.2)	SR: Frequency doing sports/ activities that are moderately energetic: every day, more than 1x/week, 1x/week, 1-3x /m, never.	LTPA		Loneliness question from Center for Epidemiologic Studies Depression Scale (CES-D) [94].	Loneliness	NA
Vance [82] USA	XS 158	52.5% male [65-91 years] 74.8 (5.8)	SR: PA Questionnaire	All types of LTPA separated		Lubben Social Networks Scale [91]	Social engagement (General SS)	
Wilcox [78] Rural USA	XS 102	All female [50+ years] M = 70.6	SR: PASE; [110]	LTPA, OPA, HHPA, (summed)		SS for ex. scale [42] (15 friends, 5 family)	SSPA	Family and friends. Average of 2 used or for analysis (overall SS)
Yeom [79] USA.	Quasi-exp. N = 64 n (IG) =33 n (CG) =31	23.4% male [60-89 years] 71 (7.4)	SR: Participation in any type of regular PA for a minimum 30 min 3x /week? (y/n)	LTPA (flexibility, balance, walking)	Answered ‘Yes’ to PA question	SS for ex. scale [42] 9 items friends and 9 family. Not clear exactly which questions.	SSPA	Friends and family (separate)

^1^ XS = cross sectional, Exp. = experimental, N = total number of participants, n = number of participants in a group, ex. = exercise, IG = intervention group, CG = control group

^2^ SR = Self-Report, /week = per week, /m = per month

^3^ LTPA = Leisure time PA, TPA = Active Transport, HPA = Household PA, OPA = Occupational PA, MVPA = moderate- vigorous PA

^4^ /day = per day, /week = per week, /m = per month, mod. = moderate, vig. = vigourous

^5^ ex. = exercise, qs = questions

### Quality rating

The quality of the four studies examining the association between general SS and PA levels was moderate for three studies [80, 81, 83] and weak for one study [82]. Ten of the 17 papers examining the association between PA specific SS and PA levels were of moderate quality and the other seven were of weak quality.

Of the six studies examining the association between loneliness and PA, all were of moderate quality, except for the longitudinal study by Newall et al. [60], which was of weak quality. For further details see Table 2.

### Measurement and analysis of social support and physical activity

The way PA was measured and analysed varied widely between studies. Overall, 23 of the studies used self-report PA measures and four used objective PA measures. The majority (74%) of studies collected continuous PA data and the remaining seven collected categorical (ordinal) data. Seven of the papers transformed the data into categories such as active or inactive based on pre-defined cut-offs for analysis purposes. Further details about collection and analysis of the PA measures in each of the three categories (i.e. general SS, SS for PA, loneliness) is described in Table 2 and Additional file 2. There were also a wide range of social support and loneliness scales used in the studies in this review. The SSPA category of studies had the most consistency of scales, with 14 out of 17 utilising various versions of The Sallis SS for Leisure Scale [42]. See Table 2 for more detail of scales used in the included studies and Additional file 2 for more detail.

### Relationship between SS, loneliness and PA

Overall, of the 21 studies examining the association between either general or PA specific SS and PA levels, 13 found a significant positive association and one study found a significant negative association [75]. Four of the six loneliness studies [58, 59, 61, 63] found a significant negative association.

In the four female only studies, one found a significant association for SSPA overall [72] and one found an association for SSPA from family only [65]. In the four studies stratified for gender, two found an association for females only. One was for general SS [80] and the other was for loneliness [59]. Of the remaining two studies, which both focused on SSPA, one found a positive association for SSPA from family but not friends in both males and females [70]. See below for further details within each of the three categories.

### General social support and physical activity levels

Five associations were reported in the four studies examining the association between generalised SS or social engagement and PA levels. Only two of these (40%) were positive and significant (greater SS being associated with greater likelihood of doing PA); one for both genders [83] and one for females only [80]. This suggests an overall unclear association between general SS and PA levels when using pre-defined cut-offs established by Sallis [57]. See Table 4 for details.

### Social support specific to PA

Of these 17 studies, 11 described at least one significant positive association between SS for PA and PA levels. Of the eight studies where source of support was not delineated, five associations (63%) were found to be positive and significant. This suggests an overall positive association between PA levels and SSPA from all sources [57]. In the eight studies where the association between PA levels and SS from friends or family were examined separately, four (50%) reported a positive association for SSPA from friends [68, 74, 76, 79] and five (63%) found a positive association for SSPA from family. One study found a negative association between SS from family and PA [75]. The one study examining SSPA from an exercise group did not find any direct association between SSPA and PA levels [71]. These results suggest an overall positive association between PA levels and SSPA from family but not from friends [57]. See Table 4 for details. When low quality studies were removed from the synthesis, the trend above was further supported, with four of the five (80%) relevant moderate quality studies finding a positive association between SSPA from family members [65, 68, 74, 79] and an indeterminate overall association between PA levels and SSPA from friends. For more detail of these results, see Tables 3 and 4.

**Table 3:** Results of association between SS and PA and quality rating

Primary Author (Year)	Type of SS^1^	Theory^2^	Type of analysis^3^	Adjustments	Results of association between PA and SS or loneliness^4^	Summary result^5^	Paper quality rating^6^
Booth [64]	SSPA	SCT [19] with comments on determinants for older adults from [112]	Forced entry logistic regression analysis	Age, sex, country of birth, marital status, employment status, living situation	Sig. greater number of active people had high social support (42.7% inactive Vs 55.6% active had high social support (*P* = 0.010). Partner or friends being active was sig. associated with being active.	+	Mod
Bopp [65]	SSPA	NS	Bivariate associations. Logistic regression analysis	nil	Sig. positive correlation between total social support (family) and strength training (ST) participation (β =1.10, *p* = 0.001) and also hours per week of ST training (β = 0.26, *p* = 0.003). No sig. association SS (friends) and ST	+ (family) 0 friends)	Mod
Carlson [31]	SSPA	SEM	Mixed Effect regression models	*Total PA* - age, ethnicity and gender, *walk for transport* : age ethnicity, months at address, number of vehicles per adult, *walk for leisure: e*thnicity and months at address	SS sig. associated with total MVPA (min/ week). B = 14.35, *p* < 0.01). SS sig. associated with min/ week walking for transport (B = 7.35, *p* < 0.05). SS sig. associated with min/ week walking for leisure (P < 0.05)	+	Mod
Gellert [66]	SSPA	NS	ANOVA. Regression analysis using MODPROBE macro	gender, age	Mean PA (F (2, 299) = 4.39, *p* < 0.05) as well as SS (F (2, 299) =5.49, *p* < 0.01) was higher in the group with individuals whose partners took part in the intervention, compared to the other two groups.	+	Weak
Hall [67]	SSPA	Socioecological model [113]	MANOVAs	age	No Sig. difference between SSPA (friends or family) on whether participants did <10,000 steps or > = 10,000 steps per day. SSE Friends: F = 0.02, *p* = 0.88 SSE Family: F = 0.02, *p* = 0.89.	0 (friends or family)	Mod
Kaplan [80]	General SS	NS	Bivariate relationships	Gender, age, education, marital status, smoking, chronic conditions, BMI, injury, functional limitations, distress, region	Higher social support was sig. associated with greater odds of doing frequent PA in females. Females OR (95%CI) =1.08 (1.04-1.13), not significant for males. OR (95%CI) = 1.04 (0.99-1.09)	+ female) 0 (males)	Mod
Kim [68]	SSPA	SEM [114]	Correlation followed by Stepwise multiple regression analysis	gender, education level, living situation, self-efficacy	SS (family) pos. associated with amount of PA Fchange (2,279) =10.24, p = 0.012 (second most important contributor to PA after self-efficacy) No sig. effect of SS from friends.	+ (family) +(friend)	Mod
Kraithaworn [69]	SSPA	Health promotion model, socio-ecological model.	Path analysis using LISREL	Nil	SS did not significantly predict PA levels directly or indirectly. (Direct effect B = 0.1. Indirect effect B = 0.08, total effect B = 0.18) SS had an indirect effect on PA levels through sense of community.	0	Mod
Lian [70]	SSPA	PRECEDE health promotion framework	Multiple regression by stepwise method	Nil	More Family encouragement and higher proportion of family members exercising was significantly associated with greater frequency of at least 20 min of moderate to vigorous exercise per week: standardised β = 0.131 and standardised β = 0.108 respectively for men and standardised β = 0.154 and 0.138 for women. (For all *P* < 0.001) In addition, frequency of contact with people significantly associated with greater frequency of moderate to vigorous exercise per week in women. Standardised β = 0.052, *p* < 0.05). No association for friends	+ (family) 0 (friends)	Weak
Luo [58]	Loneliness	NS	Cross-lagged path analysis	Age, gender, ethnicity, residence (urban or rural), education, financial independence, relative economic status, number of visiting children in 2002.	Regular PA participation decreased odds of being lonely 3 years later and loneliness decreases odds of being active in 3 years. Lonely02-- > PE05 and lonely05-- > PE08 β = -0.028, *P* < 0.001. PE02-- > lonely05 and PE05-- > lonely08. B = -0.111, *p* > 0.001.	-	Mod
McAuley [25, 71]	SSPA.	SCT (but also testing theoretical models).	Structural equation modeling	Nil	Those who reported more frequent PA, had higher levels of SS, which influenced both a better exercise experience and directly and indirectly a higher self-efficacy, which predicted higher exercise participation at both 6 and 18 months.” Model fit statistics: *χ* ^2^ (6) = 5.20, *P* > .10; NNFI, 1.0; CFI, 1.0; RMSEA, 0.027	0 (*Indirect* + associations: SS → Affect → SE → PASE 6 months → PASE 18 months)	Weak
Mowen [81]	General SS	Stress-buffering and main effect of SS on health.	Path analysis	Nil	Larger SS network size or SS satisfaction did not increase odds of having a moderate or vigorously active lifestyle. SS network β = 0.014, SS satisfaction β = 0.007	0	Mod
Netz [59]	Loneliness	NS.	ANOVA with Chi2 test and Multinomial stepwise logistic regressions	BMI, being religious versus secular, Self-rated health and education	No assoc. between odds of feeling lonely and PA level in men. In women it explained 20% of variance. Greater loneliness was associated with lower odds of engaging in sufficient PA as compared to "inactive" OR (SE) = -0.52 (0.23). Adjusted OR (CI) 0.59 (0.38, 0.94). No significant association between living alone and activity levels.	-(female) 0 (males)	Mod
Newall et al. [60]	Loneliness	Fredrickson 's Broaden and Build Theory [115]	Regression analysis	Age, gender, income satisfaction, marital status, functional status, health status	Loneliness was not significantly associated with mean everyday PA (β = 0.001, p > 0.05). Also no interaction between loneliness and happiness (B = 0.08, p > 0.05). However, greater loneliness was associated with subjectively feeling less physically active compared to peers.	0	weak
O'Brien Cousins [72]	SSPA	Theory of Planned behaviour [116] SCT [117]	Multiple regression analysis	Education, marital status, employment status, country of origin	Exercise level (more PA per week) was associated with a greater composite SSPA. B = 0.264, SE = 0.055 (*P* < 0.01)	+	Mod
Oka. [73]	SSPA.	NS.	Chi2 analysis and an independent group *t*-test	Age, gender, marital status, BMI, smoking status, alcohol consumption, self-efficacy for exercise, advice from HCP, perceived neighbourhood environment	Greater SS did not increase the likelihood of meeting PA guidelines in either males or females. Adjusted Odd Ratio (AOR) for meeting national PA guidelines and having higher SS for exercise: AOR (95%CI) = 0.82 (0.63-1.07)	0 (males or females)	Mod
Orsega-Smith [33, 74]	SSPA	SCT	Correlation analysis, multiple regression analysis and ANCOVA	Age, physical health	More LTPA significantly associated with higher SSPA from both family and friends. SS (family) Adj. B = 0.72, *p* < 0.05, SS (friends) Adj. B = 0.113, *p* < 0.0001. Also, people who met the CDC recommended guidelines for PA were significantly more likely to have higher SS from friends and family.	+ (friends) + (family)	Mod
Park [75]	SSPA	NS	Multiple regression, independent 2 sample *t*-test for high vs low-active and SS	Nil	Multiple regression. No significant correlation between SS from friends and PA. Negative association between SS family and PA. SS family: B = -0.220, t = -3.107 *p* < 0.01. Both High and low active individuals scored low on SS from friends and family with no significant difference between them.	0 (friends) - (family)	Weak
Potts [83]	General SS	Health belief model (Becker, 1974 [118, 119]	Ordinary least squares regression	Demographic factors (gender, age, education, marriage, income), health status, perceived frailty	People with stronger social support networks more likely to exercise regularly. B = 0.11 (*p* < 0.01).	+	Mod
Sasidharan [76]	SSPA	SCT	Separate factor analyses for friends and family SS	Nil	Sig. positive association between SS (friends) and LTPA b (unstandardized) (SE) = 0.13 (0.09), *p* < 0.05 No significant association for family SSL. B (SE) = -0.05 (0.03).	+(friends) 0 (family)	Weak
Schuster [77]	SSPA	SCT	Hierarchical multiple regression	Perceived barriers	Perceived SS was significantly correlated with LTPA (*r* = 0.474, *p* < 0.0001). Perceived SS accounted for an additional 17.5% of the variance in intentional exercise (*P* < 0.001)' after perceived barriers had been entered into the model.	+	weak
Shankar [61]	Loneliness	NS	Multinomial logistic regression	Age, gender, limiting long-standing illness, depression, and marital status-adjusted wealth	Loneliness associated with a greater likelihood of being inactive. OR (95% CI) reference = risky behaviour. Loneliness OR = 1.08 (1.04-1.113) of being inactive vs active. Social isolation: 1.115 (1.11-1.19) of being active vs inactive	-	Mod
Shiovitz-Ezra [62]	Loneliness	NS	Multivariate logistic regression	Age, gender, education, income, ethnicity, self-rated health. Functional impairment	No sig. association between being lonely to some degree and doing any PA. PA OR (SE) [95% CI]: 0.8 (0.11) [0.6-1.07]	0	Mod
Theeke [63]	Loneliness	NS	Chi-square statistics and one-way analysis of variance	Marital status, self-reported health, education, functional impairment, number of chronic illnesses, age, annual household income, number of individuals in household.	Chi-squared testing showed significant difference in frequency of moderate activity in Never lonely, briefly lonely and chronically lonely groups. Chi2 = 438.347 (*P* < 0.005). The chronically lonely group did less average exercise than the briefly lonely or never lonely groups (no statistical test reported for this).	-	Mod
Vance [82]	General SS	NS	Correlation and step-wise regression	Nil	No significant association between social network and total PA (*r* = 0.02)	0	Weak
Wilcox [78]	SSPA	SCT	Hierarchical regression analysis	Sociodemographic measures (age, race, education, marital status),	Non-significant trend for greater social support from friends and family (total) to be associated with higher levels of PA. B = 0.16, *p* = 0.09. Qualitative discussion identified social support as being a very common motivator to PA.	0	Mod
Yeom [79]	SSPA	Wellness Motivation Theory [120]	Repeated measures ANOVA and Chi-Square test	Nil	Intervention group (IG) significantly increased support from family (F = 21.87, p < 0.01) and friends (F = 24.72, p < 0.001) compared to controls. IG more likely to engage in regular PA after the intervention, compared with controls. Chi-squared =25.01, p < 0.001.	+ (friends) + (family)	Mod

^1^SSPA = Social Support for PA

^2^NS = Not specified. SCT = Social Cognitive Theory

^3^ANOVA = Analysis of Variance

^4^Sig. = Significant, CI = confidence interval

^5^+ = positive association, - = negative association, 0 = no association

^6^Mod = Moderate quality

**Table 4:**
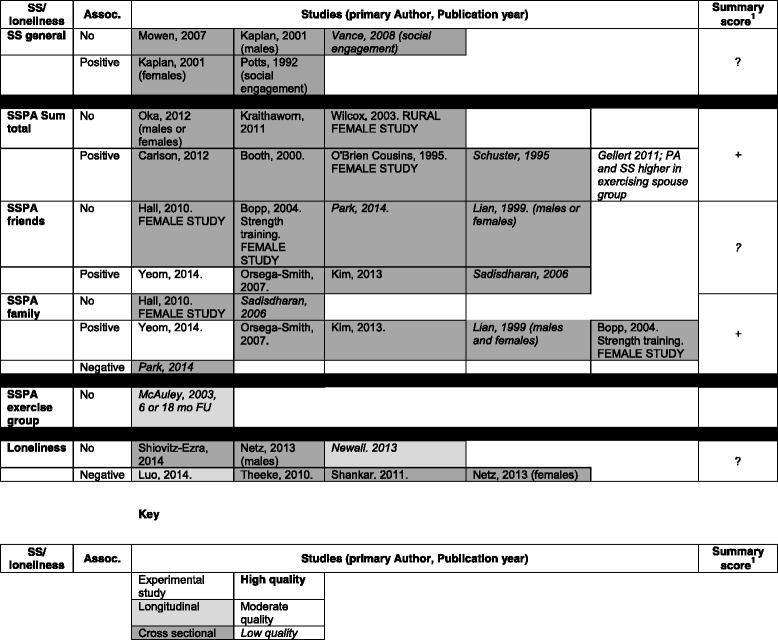
Data synthesis - summary of results

1. 0 = No association (0%-33% of the findings supported the association), ? = indeterminate association (34-59% of the findings supported the positive or negative association), + = positive association, - = negative association; (60%-100% of the findings supported the association) [57]

### Loneliness and PA levels

Seven associations were examined in the six papers focusing on the association between loneliness and PA. Four (57%) of these were significant and negative [58, 59, 61, 63], indicating unclear support for the association between loneliness and PA levels [57]. When the one low quality study [60] was removed from the synthesis, the results suggest an overall negative association with 67% being significant and negative. See Tables 3 and 4 for details

### Association between SS, loneliness and specific PA domains

Only LTPA and transport domains were assessed separately in the studies reviewed. LTPA was assessed in 15 studies and transport in one [31]. The other studies either assessed all, or a combination of several PA domains together. When placed into the three categories, the following overall trends were seen for LTPA: Three general SS studies examined LTPA [80, 82, 83]. One found a significant positive association between LTPA and SS, and one supported a significant positive association in females only [80]. This suggests an overall positive association between general SS and LTPA for females but not males [57]. Eight of the 17 SSPA studies assessed the LTPA domain. Two of these assessed SS from friends and family combined and both found significant positive associations, suggesting an overall positive association between LTPA and all sources of SS [57]. In the six studies that assessed the association between different sources of SS and LTPA, 67% of results supported a significant positive association for friends and 83% for family. This indicates an overall positive association between LTPA levels and SSPA from both friends and family [57]. Four loneliness studies examined LTPA [58, 59, 62, 63]. Within these, the results support an overall negative association between LTPA and loneliness for females only, with three of four associations [58, 59, 63] being negative and significant in females but only two of four in males [58, 63]. See Table 3 for more details. In the one study where transport PA was assessed separately, a positive association with SSPA was found [31].

## Discussion

The aims of this review were to summarise the results of quantitative studies assessing whether SS or loneliness is associated with physical activity levels in older adults. Specifically, we investigated whether any potential associations differ between *types* (e.g. task specific support, general support) or *sources* of support (family or friends or exercise group); and whether any associations between SS and PA are specific to certain PA *domains* (e.g. LTPA, transport, household, occupational). SS is an important determinant of health, especially in older adults, as there are many important life events such as retirement, illness, and death causing SS to change in later life. Understanding how SS and loneliness are associated with PA in this population group may assist in development of more effective, targeted PA interventions. This is the first review summarising the evidence in older adults.

### Relationship between different types and sources of social support, loneliness and physical activity

Because of the differences in study designs and measurement of PA and SS, it was difficult to come to a clear consensus about differences in associations between types of SS and PA. However, the following advisory assessments can be made of associations between SS, loneliness and PA in older adults. There is moderate support that higher SS specific to PA from all sources combined, and from family specifically, is associated with higher levels of PA or meeting PA guidelines. This implies that older individuals with greater support to undertake PA, specifically from their family, will be more likely to be physically active in general. An unclear association was seen for SSPA from friends and PA levels but this relationship was clarified when leisure PA was examined alone (see results below). No clear overall association was supported for general support or loneliness, but after excluding the low quality studies from synthesis, moderate quality studies did suggest a significant negative association between loneliness and PA levels, indicating that people who were more lonely had lower PA levels. Given that there were far fewer studies in these two categories, further research would be warranted to confirm these above suggestions.

### Association between SS, loneliness and specific PA domains

LTPA was the only domain examined in multiple studies. When these studies were synthesised, general SS in females and SSPA from friends and family were consistently positively associated with LTPA, rather than just family, as was evident with all studies combined. LTPA was also consistently negatively associated with loneliness in females. These findings are in line with those of a systematic review in adolescents, where more consistent positive associations were found between SS from all sources and LTPA levels than when PA domains were not separated [51]. The authors of the above review noted that questions in the SSPA scales focused on provision of SS for leisure and sport PA, which may explain why there were less consistent associations when other domains of PA were included in the analysis. The same is likely to be true for the Sallis SSPA scale used in many of the papers in this study, as all the questions are focused around exercising specifically [42]. Older adults are unlikely to associate other domains of PA (e.g. household or transport) as forms of ‘exercise’ and thus are likely to exclude this PA when considering the questions in the scale. Additionally, there is a difference between leisure activities and other types of physical activity (such as house work, transport or employment) in that they are done for solely enjoyment rather than function [84]. Social interaction and enjoyment have been described as two key reasons for participating in sport or (leisure) physical activity in both children and adults [85]. In adults, emotional support from others has been found to be positively associated with intrinsic motivation for PA (“behaviour engaged for pleasure and enjoyment”; p37 [47]) and in turn, participation in moderate to vigorous PA and walking. This suggests that greater emotional support from others encourages greater enjoyment in physical activity, which in turn makes people feel more motivated to do leisure exercise [47]. It is, however, less likely that greater support will likely have any impact on transport, occupational or household PA. To further explore potential differences between SS and different PA domains, future studies would benefit from using more detailed PA measures (either accelerometers or detailed scales) and ensuring domain specific PA is assessed as well as total PA.

### Other general findings of the review

There were some gender differences highlighted in this review. Of the four studies that stratified by gender, there was some suggestion that the PA levels of women are more likely than men to be influenced by general SS [80] or loneliness [59], but not by SSPA [35, 70]. Social support has been positively associated with self-rated health in older women but not men in a number of studies [86, 87]. Sex differences could exist in the association between SS or loneliness and PA as well, as PA is a health-related variable. Given the small number of studies exploring this association and the sex differences in the typical ageing process, with women more likely to live longer with more health conditions than men [1], this warrants further investigation.

Most studies in this review used a very generic definition for older adults, for example everyone aged 60+ years, and did not stratify by age in the analysis. However, in reality, there are great differences in life circumstances between people who are defined as young old, mid-old and older old. Social factors like social support may influence PA levels differently amongst those in different age groups, with different health statuses, and in response to different life events. For example, a longitudinal study of women found that the death of a spouse was associated with increased PA in women aged 55-60 years but the same event had no impact on the PA levels of women aged 70+ years [88]. Although the age range of people who retire is broadening, the typical retirement age is 65 years and retirement is therefore more likely to be associated with PA in people in their mid-sixties than other age groups. Retirement has indeed been positively associated with PA levels in middle aged women [88]. Therefore, further associations may have been seen in some studies if they had been stratified by age or life stage. It may be worthwhile for researchers to consider doing this in future studies to examine if the association differs between subgroups of ‘older people’.

All studies in this review were rated as weak to moderate for their measures of the key variables SS and PA (see Table 1). Part of the reason for the low quality rating of the PA measures in particular was that a decision was made by the authors to include papers with adequate face validity of these measures. This was decided because the aim of this review was to provide an overview of the association between SS and PA in a variety of studies; individualised, detailed assessments of questionnaire quality were outside its’ scope. Face validity was deemed an appropriate measure of validity, as it is important and relevant for all study designs and purpose [54]. The use of validated PA measures was included in the quality rating procedure and therefore studies without validated PA measures were rated as low quality. Less than half the studies used externally validated PA scales or objective PA measures. This was probably because the majority of these studies did not specifically aim to examine the association between SS and PA. Thus, these constructs were often measured as part of a large test battery, which included brief measures of PA rather than more extensive validated questionnaires or objective measurements. However, the sample sizes of these studies were all greater than 900, and in large studies these more generalised questions amongst large test batteries are more acceptable [89].

### Study limitations and strengths

This systematic review has several limitations. Firstly, there are limitations with regards to the inclusion of studies. Studies in older adults with specific medical conditions, such as obesity, cancer, heart conditions or other chronic illnesses, mental illness or disabilities were not included. This is likely to have excluded a number of potentially relevant studies because many older adults do have chronic illnesses and much research has been conducted with clinical populations. However, these studies were excluded due to the likelihood that SS relevant to clinical populations may differ to that more prevalent in the general population. In addition, qualitative studies, which can often offer more insight into a topic, were not included in this review to make comparison of studies more direct.

The variability of outcome measures used for assessing PA and SS or loneliness also made comparison of study results difficult. Specifically, there was almost no cross-over between the types of SS scales used in the general SS studies, with only two of these SS studies using the same SS scale, the Lubben Social Network Scale [82, 83]. Scales measured one or a combination of the following: perception of support available, number of people available to provide support and satisfaction with support, indicating different components of SS that may have different associations with PA. There was also inconsistency in the measurement of SS for PA, with only one study [78] using the original scale developed by Sallis et al. [42] and 13 others using various versions of it. The original validation study had been conducted in people under the age of 45 [42], thus it may not be appropriate to measure SSPA in older people. The remaining three SSPA studies used different SSPA measures altogether [71–73]. Therefore, it is difficult to compare these studies conclusively. However, these SS measures do share some similar items such as family and/or friends offering verbal encouragement to do PA, or exercising together. The loneliness studies had much more overlap in loneliness measures, with three studies using the CES-D one item scale, assessing how often people felt lonely in the past week, and two of the other studies used scales with similar wording [58, 61]. While there were differences in the scales used, there was greater agreement in the way SSPA and loneliness were assessed, than for general SS. This implies that the overall findings for the associations between SSPA and loneliness and PA are more reliable, but the general SS measures varied too much to have a strong sense of the overall association.

Despite the above limitations, inclusion of different study designs and studies with a variety of PA measures in the review has provided a detailed overview of current knowledge about SS and PA in older adults. Use of a quality rating scale suitable for different designs has allowed the authors to differentiate studies of differing quality and make stronger assumptions about the overall association between SS and PA. Use of the quality rating scale has also highlighted a starting point for future research.

### Future research

This review highlights a need for research with regards to measures of SS and PA in older people. The population is ageing at an unprecedented rate and as both SS and PA are key determinants of healthy ageing, it is important to develop and validate a general SS scale specific for older adults to be used consistently across studies examining factors associated with healthy ageing. Further validation in older adults of the Sallis SS for Exercise Scale [42], and consistent future use of this scale would also simplify and strengthen cross-study comparisons. Furthermore, the use of one well-validated PA scale which allows assessment of all modes of PA to be assessed but also be analysed separately would help inform whether other domains of PA are as influenced by SS as is the lifestyle domain.

Given the lack and variation of research available investigating the role of general support for PA levels in older adults firm conclusions were not able to be made in that category. But given the value of social support for the health and wellbeing of older adults, future research specifically in this area would be warranted.

The body of evidence for SSPA and PA was greater but nearly all these studies were cross-sectional. Therefore, it is not possible to make statements about the direction of the association, higher SSPA could be associated with higher PA, but it may also be the other way around. This field of research would therefore benefit from prospective or longitudinal studies assessing associations between SSPA and PA over time. Natural experiments could also help to elucidate the prospective association, for example, by observing the impact of joining sporting clubs or community groups offering PA options for older adults on PA levels and SSPA. There would also be great benefit in performing intervention studies where social support is manipulated to examine if changes in social support result in increased physical activity levels in older adults.

## Conclusions and implications

Notwithstanding the large variability in study methodologies, in general it seems SS specific to PA is an important factor assisting older adults to be physically active, especially when coming from family members. The evidence also highlights the importance of friend support for leisure time PA in older adults. In terms of general SS, there does not seem to be an association with PA, however with far fewer studies investigating this relationship, more studies are needed to either confirm or challenge this finding. Finally, the moderate quality loneliness studies suggest a negative association between loneliness and PA levels, especially in females.

The findings from this review suggest that PA interventions for older adults should specifically take into consideration family as important sources of SS for general PA promotion that aims to increase PA levels across a number of PA domains. Additionally, the importance of friends as sources of support for leisure time PA in older adults is highlighted here. ‘Buddy’ style interventions where participants are encouraged to exercise with a partner have been successful in the general population [32] and in older adults [90]. This review suggests that this type of intervention may benefit from targeting family members as buddies or to be otherwise involved in the intervention. Finally, generalised support in the lives of older adults, as well as loneliness, may also significantly influence leisure-time PA participation, especially in women. As such, the promotion of the social benefits of PA participation should be part of interventions aimed at older adults.

## Additional files

Additional file 1: PubMed search strategy. (DOCX 16 kb)

Additional file 2: Further detail about PA measurement and analysis. (DOCX 13 kb)

## Abbreviations

LTPA: Leisure time physical activity
MVPA: Moderate to vigorous physical activity
PA: Physical activity
SS: Social support
SSPA: Social support for physical activity
