# Supplementary material for: The association between social support and physical activity in older adults: a systematic review
Source: Int J Behav Nutr Phys Act. 2017 Apr 27;14:56. doi: 10.1186/s12966-017-0509-8 (PMC5408452; doi:10.1186/s12966-017-0509-8)
Supplement: Supplementary file 1 — PubMed search strategy. (DOCX 16 kb) [file 12966_2017_509_MOESM1_ESM.docx]

**Additional file 1.**

**PubMed search strategy**

**Searched 14 August at 9.30am -1775 results**

**Search terms**

**For social support:**

1. (social support [MeSH])
2. (“social support” [tiab])
3. (“social connect*”[tiab])
4. (“social network*” [tiab]))))

For loneliness

1. ((loneliness [MeSH])
2. (loneliness [tiab])
3. (“social isolat*” [tiab])

For Physical activity

1. (motor activity [MeSH])
2. (“Physical activit*” [tiab])
3. (exercise [tiab])
4. (Exercise, physical [MeSH])
5. (Sport [tiab])
6. (walk* [tiab])

For population

1. (adult [MeSH])
2. (adult [tiab])
3. (elderly [tiab])
4. (seniors [tiab])
5. (child* [tiab])

Combining

1. 1 OR 2 OR 3 OR 4 OR 5 OR 6 OR 7
2. 8 OR 9 OR 10 OR 11 OR 12 OR 13
3. 14 OR 15 OR 16 OR 17 NOT 18
4. 19 AND 20 AND 21

*Captures alternative word endings
