# Supplementary material for: The association between social support and physical activity in older adults: a systematic review
Source: Int J Behav Nutr Phys Act. 2017 Apr 27;14:56. doi: 10.1186/s12966-017-0509-8 (PMC5408452; doi:10.1186/s12966-017-0509-8)
Supplement: Supplementary file 2 — Further detail about PA measurement and analysis. (DOCX 13 kb) [file 12966_2017_509_MOESM2_ESM.docx]

**Additional File 2:**

**Further detail about PA measurement and analysis**

Three of the four general SS studies collected continuous PA data [80, 83, 82] and these data were dichotomised for analysis in one study [80]. The remaining study collected categorical (ordinal) data in a single item survey [81].

Of the 17 studies examining the association between PA specific SS (SSPA) and PA, 11 collected self-reported continuous PA data and three collected continuous objective data [67, 31, 73]. Most studies analysed the data in the manner it was collected. However, in four of these 14 studies [64, 73, 70, 67] data were dichotomised for analyses using predefined cut-offs such as government recommended PA guidelines (e.g. Regular vs irregular PA or active vs inactive). Two studies analysed both continuous and dichotomous data [74, 75]. The remaining three SSPA studies collected self-report categorical PA data and analysed these as dichotomous [79], ordinal [77], or continuous [69] variables.

Of the six studies examining the association between loneliness and PA, one objectively measured continuous PA data [60] and two collected self-report continuous data, either categorising [59] or dichotomising [61] them for analysis. The three remaining loneliness studies collected categorical PA data [63, 58, 62]. One of these analysed dichotomised data for analysis [62]. See Tables two and three for further detail about the way PA was assessed and analysed in the included studies.

**Further detail about SS scales**

The Lubben Social Engagement Scale [84] was used in two of the four studies on general SS and PA [83 , 82]. The Social Support Questionnaire (SSQ [85]) and a perceived social support scale [86] were used in the remaining two studies on general SS and PA. The Sallis SS for Leisure Scale [42] was the most commonly utilised SSPA scale, used in 14 of the 17 SSPA studies. One study used the full scale [78] and the other 13 studies used some variation on the original scale with between two and 24 questions, with two of these translating the scale into other languages [68, 69]. Of the 17 studies examining the association between SS specific to PA, eight assessed a total score, reflecting all SSPA sources in the participants’ life, eight assessed SSPA from friends and family separately and one intervention study measured SSPA provided by an exercise group [71]. The most commonly used loneliness scale was the one item loneliness questionnaire from the Center for Epidemiologic Studies Depression Scale [87], utilised in three of the studies [62, 63, 59].
